# Gene Sequence Variability of the Three Surface Proteins of Human Respiratory Syncytial Virus (HRSV) in Texas

**DOI:** 10.1371/journal.pone.0090786

**Published:** 2014-03-13

**Authors:** Lorena I. Tapia, Chad A. Shaw, Letisha O. Aideyan, Alan M. Jewell, Brian C. Dawson, Taha R. Haq, Pedro A. Piedra

**Affiliations:** 1 Department of Molecular Virology and Microbiology, Baylor College of Medicine, Houston, Texas, United States of America; 2 Programa de Virología, Instituto de Ciencias Biomédicas, Facultad de Medicina, Universidad de Chile, Santiago, Chile; 3 Departamento de Pediatría y Cirugía Infantil Norte, Facultad de Medicina, Universidad de Chile, Santiago, Chile; 4 Department of Molecular and Human Genetics, Baylor College of Medicine, Houston, Texas, United States of America; 5 Medicine School, Baylor College of Medicine, Houston, Texas, United States of America; 6 Department of Pediatrics, Baylor College of Medicine, Houston, Texas, United States of America; University of Iowa, United States of America

## Abstract

Human respiratory syncytial virus (HRSV) has three surface glycoproteins: small hydrophobic (SH), attachment (G) and fusion (F), encoded by three consecutive genes (SH-G-F). A 270-nt fragment of the G gene is used to genotype HRSV isolates. This study genotyped and investigated the variability of the gene and amino acid sequences of the three surface proteins of HRSV strains collected from 1987 to 2005 from one center. Sixty original clinical isolates and 5 prototype strains were analyzed. Sequences containing SH, F and G genes were generated, and multiple alignments and phylogenetic trees were analyzed. Genetic variability by protein domains comparing virus genotypes was assessed. Complete sequences of the SH-G-F genes were obtained for all 65 samples: HRSV-A = 35; HRSV-B = 30. In group A strains, genotypes GA5 and GA2 were predominant. For HRSV-B strains, the genotype GB4 was predominant from 1992 to 1994 and only genotype BA viruses were detected in 2004–2005. Different genetic variability at nucleotide level was detected between the genes, with G gene being the most variable and the highest variability detected in the 270-nt G fragment that is frequently used to genotype the virus. High variability (>10%) was also detected in the signal peptide and transmembrane domains of the F gene of HRSV A strains. Variability among the HRSV strains resulting in non-synonymous changes was detected in hypervariable domains of G protein, the signal peptide of the F protein, a not previously defined domain in the F protein, and the antigenic site Ø in the pre-fusion F. Divergent trends were observed between HRSV -A and -B groups for some functional domains. A diverse population of HRSV -A and -B genotypes circulated in Houston during an 18 year period. We hypothesize that diverse sequence variation of the surface protein genes provide HRSV strains a survival advantage in a partially immune-protected community.

## Introduction

Human respiratory syncytial virus (HRSV) is the major cause of lower respiratory disease among infants [1], [2], a frequent pathogen in elderly and immunosuppressed patients [3] and a major public health concern worldwide [4]. Although an immunoprophylactic approach [5] has been approved for infants with high-risk medical conditions (palivizumab or Synagis®) [6], no safe and effective vaccine has been licensed to date for use in humans.

As a member of the family *Paramyxoviridae*, HRSV is a nonsegmented negative-strand RNA virus [7]. The viral genome of ∼15,200 bases contains 10 genes that are transcribed to 11 proteins in a 3′ to 5′ sequential order [7]. The small hydrophobic (SH), the attachment (G) and the fusion (F) genes code the three surface proteins of the virus. The variations on monoclonal antibody binding patterns, driven by the antigenic diversity on the G glycoprotein [8] have led to the identification of two antigenic groups: HRSV-A and HRSV-B [9]. The G glycoprotein is a type II glycoprotein that contains two hypervariable regions flanking a non-glycosylated central conserved domain. The analysis of the nucleotide sequence on the second hypervariable region of the G gene has led to classification of HRSV genotypes within HRSV -A and -B groups [10] and revealed that multiple genotypes can co-circulate during the same epidemic season [10], [11]. Since then, many studies have used this approach to report the molecular epidemiology and genetic variability of HRSV worldwide [11]–[22]. For HRSV-A, at least ten genotypes (GA1–GA7 [10], [11], SAA1 [22], NA1 and NA2 [21]) have been described, with GA2 and GA5 predominating in many countries in recent years [13], [16], [18], [19]. Interestingly, a recent novel HRSV-A genotype with a 72-nucleotide G gene duplication was reported in Canada [23] (called genotype ON1). In the case of HRSV-B strains, phylogenetic analyses have identified at least 13 genotypes (GB1–GB4 [10], SAB1–SAB3 [12] and BA1–BA6 [17]). Similar to the ON1 genotype, a 60 nt-duplication in the G gene of HRSV-B was identified in 1998 in Buenos Aires. This novel HRSV-B subgroup was named BA genotype [24], and spread worldwide within a 7 years period, replacing the other prevailing HRSV-B genotypes by 2005. It is remarkable that neither ON1 nor BA genotypes have been associated with a different virulence profile, even though major changes occurred in the distal fragment of the G gene. Moreover, no consistent association has been established between severity of disease and HRSV genotype.

Recently, new evidence has shown that the three surface proteins of HRSV, - G, F and SH - have relevant roles in pathogenesis and the host immune response. The G protein is involved in the viral attachment [25] through heparan sulfate-like moieties (proteoglycans) on the apical surface of epithelial cells [26]. The extensive glycosylation in the hypervariable regions of the ectodomain has shown to be relevant for virus infectivity [27], probably in relation with its complex secondary structure [8]. Nevertheless, no specific pathogenic role has been attributed to this “mucin-like” conformation. A CX3C motif has been described in the central conserved domain of the G protein, similar to the CX3C domain of the chemokine fractalkine. This motif has been shown to mimic the leukocyte chemotactic activity of fractalkine *in vitro* [28] and to inhibit the activation of the NF-κB and the secretion of inflammatory cytokines by human monocytes, suggesting an inhibitory role on host innate immune response to HRSV [29]. The secreted form of G protein (soluble G), generated by the initiation of translation at a second AUG codon of the open reading frame [30], has been shown to inhibit Toll-like Receptor (TLR) 3/4 mediated IFN-beta *in vitro* [31], suggesting that it may also play a role in suppressing the innate immune response.

The F protein, which mediates the fusion and later formation of syncytia, is assembled into trimers in the membrane surface and is modified by the addition of N-linked carbohydrates. A conserved hydrophobic fusion peptide region has been described, followed by two heptad repeat domains that are separated by a cysteine-rich region [32] shown to be important for fusion activity [33]. A cytoplasmatic tail in its C-terminal end is relevant for virion assembly [34], by the recruitment of viral proteins into filaments in the cell surface. The fusion protein plays a major role in viral attachment, interacting with the recently described HRSV receptor: nucleolin [35]. The F protein induces the innate immune response, by interacting with TLR-4 on human leukocytes [36] and possibly epithelial cells, and promoting p53-dependent apoptosis [37].

The SH, it is a highly conserved small surface protein [38], [39], with a hydrophobic core possibly corresponding to a single transmembrane domain [40]. Molecular modeling studies have suggested that pentamers or hexamers [41] are formed with a circular structure and a central pore [42]. Although it is not clear its role in HRSV life-cycle, it has been shown that deletion of the protein results in attenuation of replication in animal models [43], [44]. It has been described as a ion channel [40] mediating membrane permeability [41], and more interesting, it has been implicated in inhibition of apoptosis by the TNF-α pathway [45], [46]. However, the genetic variability of the genes encoding the three surface proteins of RSV observed through successive epidemics and their phylogenetic topologies have not been studied.

In this manuscript we describe the genetic and amino acid variation in the three major surface proteins of RSV from 60 clinical isolates collected from 1987 to 2005 from one medical center. We compare the major contemporary genotypes to the prototype genotypes detected in the late 1950s and early 1960s and provide genetic information on the domains that drive the variation on the SH, G and F surface proteins.

## Materials and Methods

### Ethics Statement

HRSV isolates were selected from the biorepository securely stored in a limited access laboratory of the senior investigator (PAP) at the Department of Molecular Virology and Microbiology of Baylor College of Medicine. All samples stored in the biorepository are assigned a unique laboratory number and barcode, and are linked to a secured database with limited access. All nasopharyngeal aspirates, nasal wash samples, and throat swab samples were collected from consented participants who had participated in IRB approved studies at Baylor College of Medicine. Future use authorization at the time of consent was required for storing virus positive samples in the biorepository. Many of the RSV isolates reported in this study were collected before future use authorization was mandated. For this study no additional IRB approval was obtained for sequencing coded RSV isolates. Clinical data from the RSV infected subjects were not provided other than the year and city the virus was isolated.

### Virus strains

The study included sixty original clinical isolates of HRSV collected from children with lower respiratory symptoms in Houston, Texas from 1987 to 2005. The samples were selected from the biorepository at the Department of Molecular Virology and Microbiology of Baylor College of Medicine where they were stored at −80°C. Five prototype strains used in our laboratory for research were also included: RSV-A-USA-Long-56, RSV-A-TX-Tracy-Oct87, RSV-A-Bernett-61, RSV-A2-AUS-61 and RSV-B-CH-18537-62. The number at the end of the nomenclature represent the year of isolation in the twentieth century. The original clinical isolates collected in Texas were isolated on HEp-2 cells culture tubes. Viral cultures with cytopathic effect (CPE) were passed a second time in a 24-well plate plaque assay. A third passage was performed in a 25 cm^2^ flask with HEp-2 monolayer. The flask was harvested at days 3 or 4 post-inoculation when the monolayer demonstrated approximately 75% CPE. Infected cells were lysed using sterile glass beads; the supernatant was sonicated and then clarified by low speed centrifugation. The clarified supernatant was mixed with equal volume of 15% glycerol in Iscoves DMEM for stabilizing the virus during freeze/thaw conditions. Aliquots were made, snapped-frozen, and stored at −70°C for future analysis.

### Primer design

**Figure 1:**
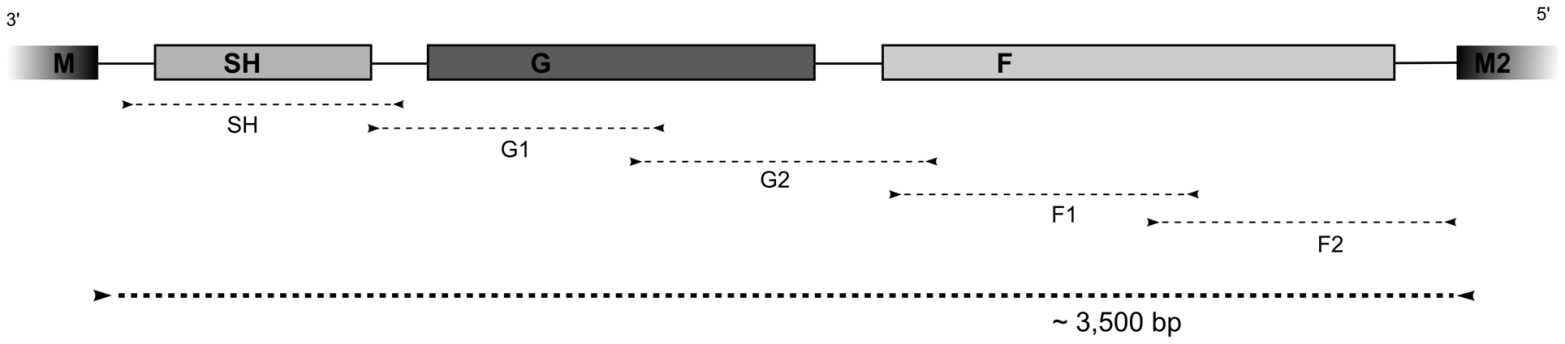
Sequencing Strategy. The region of the HRSV genome (SH-G-F) was amplified by PCR using five overlapping fragments, ranging from 547 to 1262 bp in length. Sequencing was performed in forward and reverse directions and final consensus was obtained by contig assembly for each sample.

The strategy used to amplify the region of the viral genome that includes the SH, G and F genes is illustrated in Figure 1. A previously described approach by Kim et al [47] was used to obtain the F1 region of the F gene. The rest of the primers (IDT, Iowa, USA) were designed based on the available sequence data from Genbank: U50362 (RSV A2) and NC_001781 (RSV B1). Briefly, the segment of interest on the RSV genome was amplified by PCR using five overlapping fragments of 547 to 1262 bp length, as shown in Table 1. For the fragments F1 and F2, we used the same set of primers to amplify HRSV-A and HRSV-B strains because these regions are highly conserved in both RSV groups (F1-AB-FWD/F1-AB-REV and F2-AB-FWD/F2-AB-REV). For the other regions, we used different sets of primers to amplify RSV-A and RSV-B viruses.

**Table 1:** Primers and specific conditions used for PCR amplification of RSV surface proteins genome.

Fragment	RSV Group	Primers	PCR conditions#	Fragment Length (bp)
**SH**	A	SH-A-FWD: CCC AGA TCA TCC CAA GTC AT	32 cycles:	547
		SH-AB-REV: TGT TTG GAC ATG GTT GCA TT	(94°C×30″-59°C×30″- 72°C×1′)	
	B	SH-B-FWD: CAC AAA CCA ATC CCA CTC AA	32 cycles:	571
		SH-AB-REV: TGT TTG GAC ATG GTT GCA TT	(94°C×30″-59°C×30″- 72°C×1′)	
**G1**	A	G1-A-FWD: TCA AGC AAA TTC TGG CCT TA	35 cycles:	935
		G1-A-REV: GGT TTT TTG TTG GGT ATT CTT TTG C	(94°C×30″-58°C×30″- 72°C×1′)	
	B	G1-B-FWD: ACA AGC AAA TTT TGG CCC TA	35 cycles:	874
		G1-B-REV: CAG GGA ACG AAG TTG AAC AC	(94°C×30″-58°C×30″- 72°C×1′)	
**G2**	A	G2-A-FWD: GAA GTG TTC AAC TTT GTA CC	35 cycles:	755
		G2-A-REV: CTG CAA TTC TGT TAC AGC AT	(94°C×30″-55°C×30″- 72°C×1′)	
	B	G2-B-FWD: CAC ACC ACA CAA CAG CAC AA	35 cycles:	1015
		G2-B-REV: CCC AGA AAT CTT CGT TTC CTC	(94°C×30″-55°C×30″- 72°C×1′)	
**F1**	A&B	F1-AB-FWD*: GGC AAA TAA CAA TGG AGT TG	(94°C×30″-48°C×30″- 72°C×1′)×5 cycles, then	A: 1047
		F1-AB-REV*: AAG AAA GAT ACT GAT CCT G	(94°C×30″-55°C×30″- 72°C×1′)×35 cycles	B: 1065
**F2**	A&B	F2-AB-FWD*: TCA ATG ATA TGC CTA TAA CA	(94°C×30″-48°C×30″- 72°C×1′)×5 cycles, then	A:1255
		F2-AB-REV: GGA CAT TAC AAA TAA TTA TGA C	(94°C×30″-55°C×30″- 72°C×1′)×35 cycles	B:1262

# : Mg: Primers:

*: [47].

### RNA extraction and Reverse Transcription PCR

Viral RNA was extracted from the supernatant of the third HEp-2 cell passaged RSV clinical isolates using the Mini Viral RNA Kit (Qiagen Sciences, Maryland, USA) with the automated platform QIAcube (Qiagen, Hilden, Germany) according to the manufacturer instructions. cDNA was generated by reverse transcriptase reaction using random hexanucleotide primers (New England Bio Labs, Ipswich, MA, USA) and avian myeloblastosis virus (AMV) reverse transcriptase (Life Sciences, St. Petersburg, FL, USA) at 43°C for 60 minutes in a 2720 Thermal Cycler (Life Technologies, Applied Biosystems, Carlsbad, CA). Amplification of each gene fragment was performed using Platinum ® Taq Polymerase (Invitrogen) in a 2720 Thermal Cycler. Primers and conditions of PCR reactions are shown in Table 1. PCR products were visualized by 1% agarose gel electrophoresis.

### DNA sequencing and contig construction

PCR products were purified using ExoSAP-IT reagent (Affymetrix, Inc) as per manufacturer instructions and sent for sequencing by Sanger method at Genewiz (South Plainfield, NJ). The sequencing was performed in forward and reverse directions for each gene fragment and the quality score of chromatogram traces was confirmed by visualizing them with BioEdit Sequence Alignment Editor Version 7.0.9.0 [48]. SeqMan® program of Lasergene 8 program suite (DNASTAR, Inc, Madison, WI) was used for contigs assembling and obtaining the final SH-G-F gene sequence for each clinical isolate (∼3.500 bp). The nomenclature adopted for our isolates included the RSV group (A or B), followed by the place of isolation (Texas, TX), laboratory isolate number and date of isolation (e.g., A-TX-79218-Nov 04). Finally, EditSeq of Lasergene 8 program suite was used to extract the coding sequences (CDS) of SH, G and F genes for further analysis. For the genotype analysis, a fragment of ∼270 bp located in the second hypervariable region of the G gene was obtained for each strain [10].

### Phylogenetic analysis

The phylogenetic analyses were performed with R software (R Development Core Team, 2009) using recently developed phylogenetic tools (APE [49] and phangorn [50] packages). Multiple sequence alignments were performed by CLUSTAL W for the entire SH-G-F gene fragment and CLUSTAL X (version 1.83) for CDS sequences [51]. Analyses included a likelihood based model selection seeded with the UPGMA tree to identify the most appropriate nucleotide substitution model, followed by maximum likelihood analysis of phylogeny with evolutionary distances calculation. Bootstrap analyses were performed using the bootstrap.pml method in the phangorn package with 1,000 replicates to evaluate the tree topology and group structures identified. Bootstrapping values >75% were considered significant.

The genotype distribution analysis of the clinical isolates was performed based on the methodology described by Peret et al [10] that compared the second hypervariable region of the G gene. To improve the robustness of the analysis, gene sequences of HRSV were retrieved from Genbank as references for comparison with the Houston clinical isolates (Table S1). A total of 62 partial G gene sequences of HRSV-A (270 nt) and 53 of HRSV-B (270 to 330 nt) were retrieved from Genbank. A phylogenetic analysis of SH, G and F genes (CDS) was performed in relation to genotype distribution of clinical isolates collected in Houston from 1987 to 2005.

### Genetic variability analysis

**Figure 2:**
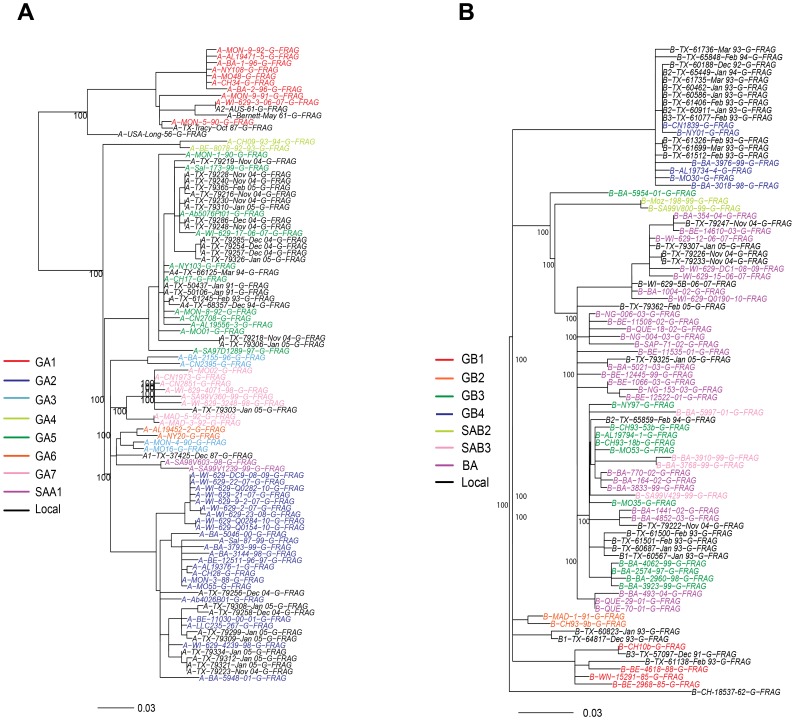
Phylogenetic trees of HRSV strains group A (A) and B (B), based on the second hypervariable region of G-gene as described by Peret et al [10] . Selected worldwide previously described sequences (color), were retrieved from GenBank and compared to local strains from Houston, TX (black). Phylogenetic tree construction was performed by maximum likelihood analysis, and bootstrap values were calculated to support clustering. Only bootstrap values greater than 75% are shown.

The variability within and between groups was examined for each site in each gene as well as cross tabulation of patterns of variation against functional regions that have been previously annotated. In the F gene there are 6 regions for which - to our knowledge - no function has been previously described. These regions in the F gene were assigned the term “not defined” and numbered sequentially from 1 to 6. At the nucleotide level, the measure of distributional divergence was determined by calculating Kullback–Leibler (KL) divergence in each site by the comparison of nucleotide distributions between groups defined by the phylogenetic analysis (i.e. the colored groups in the trees shown in Figure 2). In order to obtain information about variation of the contemporary clinical strains, the comparisons were performed between genotypes GA1 (prototype isolates) and others in the HRSV-A group and between strain B-CH-18537-62 (prototype strain) and others for HRSV-B strains. After examining the KL divergence values using kernel density plots of the values across all sites, KL values of 0.5 or more per nucleotide site were considered significant. The percentages of those divergent sites across the functional regions were calculated.

### Analysis of differences between HRSV viruses by genotypes and protein domains

The variability within and between genotypes and genes was investigated by the analysis of the rate of non-synonymous (Dn) to synonymous (Ds) variation by domains to identify the domains of viruses which appeared divergent between genotypes. This analysis was performed by utilizing the multiple sequence alignments together with strain inferences. Translated amino acid sequences were determined from the alignments, and a comparison of DNA-level variation and amino acid variation was used for analysis. Sites were considered synonymously variant when they displayed nucleotide variation but not amino acid (aa) variation between a pair of sequences. We grouped sequences into HRSV genotypes according to the phylogenetic analysis, and we tabulated the total number of non-synonymous and synonymous changes between sequences in different genotypes using all pairwise combinations of sequences from different groups. Comparisons were performed between the sequences from groups GA1 (prototype viruses), GA2 and GA5 for HRSV-A and between groups GB3, GB4 and BA (contemporary B-isolates) in the HRSV-B strains. Variation among viruses was recognized when the Dn/Ds (x) was greater than 1, while x<1 was considered to be convergent and x = 1 was a neutral selection. Functional annotation of sequence regions was superimposed on these results to reveal pattern in our statistic that correlated the Dn/Ds tabulation with prior functional knowledge of protein regions.

### Nucleotide sequence accession numbers

The sequences from Texas, USA reported in this study were deposited in GenBank database under accession numbers JX198105 to JX198169.

## Results

Nucleotide sequencing of the SH, G, and F genes of the HRSV was performed in the 60 original isolates, covering a period from 1987 to 2005. The number of original samples analyzed per epidemic period was: 1 for 1987, 3 for 1991–92, 18 for 1992–93, 5 for 1993–94, 1 for 1994–95 and 32 for 2004–05. Prototype strains from years 1956 (A-Long, USA), 1961 (A2, Australia), 1961 (A-Bernett, USA), 1962 (B-18537, USA) and 1987 (A-Tracy, TX) were also sequenced. In total, 35 HRSV-A strains and 30 HRSV-B were studied.

The analyzed genes (SH-G-F) from HRSV-A samples corresponded to nucleotides 4,171 to 7,630 in the reference strain A2 from Genbank (accession number U50362 [52]). For HRSV-B isolates, the segment corresponded to nucleotide (nt) 4,144 to 7,391 of the B1 strain in Genbank (accession number NC_001781 [53]). The total length ranged from 3,454 to 3,460 nt for HRSV-A samples, and from 3,246 to 3,307 nt for HRSV-B group. The 60-nt duplication in the G gene previously described [24], and characteristic of Buenos Aires (BA) strains was detected in 7 HRSV-B original samples from the epidemic season 2004–2005. Also a previously described six-nucleotide deletion [15] was detected in four of the BA strains (TX-B-79226-Nov04, TX-B-79233-Nov04, TX-B-79247-Nov04 and TX-B-79307-Jan05). The nucleotide comparison between the HRSV-A strains and HRSV A-Long-1956, the oldest strain sequenced, showed percentages of identity of 93.2 to 95.2%. Only one pair of samples collected during the same epidemic season had 100% identity in the SH-G-F genes (TX-A-79230-Nov04 and TX-A-79310-Jan05). The identity between HRSV-B strains and reference HRSV B-18537-1962 was 95.1 to 96.5%. Three HRSV-B samples collected during the same epidemic period had 100% identity in their SH-G-F genes (TX-B-60188-Dec92, TX-B-60462-Jan93 and TX-B3-61077-Feb93).

### Phylogenetic Trees and Genotype analysis

To determine the genotype classification of our isolates, the phylogenetic analysis was performed by comparing the last 270 nt fragment of the G gene of the HRSV isolates to those with previous genotype designations [10]–[13], [15], [16], [54], [55]. The phylogenetic trees obtained are shown in Figure 2. For HRSV-A strains (Figure 2A), two major branches were identified. The first one consisted of the previously described GA1 genotype which included all the prototype strains: A-USA-Long-56, A-TX-Tracy-Oct87, A-USA-Bernett-May61, and A2-AUS-61. A distance is present between A-USA-Long-56 strain and the rest of the GA1 isolates, supported by a bootstrap value of 100%. On the second major branch, where all the original TX samples are located except for A-TX-Tracy-Oct-87, bootstrapping values of 100% support clusters that correspond to previously described genotypes: GA2, GA4, GA5 and GA7. A clear differentiation was not observed for genotypes GA3, GA6 and SAA1 which appeared to group together. The TX samples primarily grouped with the GA5 and GA2 genotypes. Twenty of the TX HRSV-A isolates clustered with GA5 viruses, corresponding to isolates collected during years 1991, 1993, 1994, 2004 and 2005. Nine other TX HRSV-A isolates collected during the epidemic 2004–2005 clustered in the GA2 genotype. One TX HRSV-A isolate from the 2005 RSV season grouped with the GA7 genotype (A-TX-79303-Jan05), and another TX HRSV-A strain collected in 1987 clustered close to the GA3-GA6-SAA1 group (A-TX-37425-Dec87).

The distribution of HRSV-B strains is shown in Figure 2B. The prototype HRSV strain, B-CH-18537-62, is located in a separate branch distant to all other strains. Two of the TX HRSV-B isolates collected in 1991 and 1993 grouped with the GB1 genotype as supported by a bootstrap value of 100% (B3-TX-57097-Dec91 and B-TX-61138-Feb93). Clusters of GB2 and SAB2 genotypes with 100% bootstrapping values were recognized but none of the TX HRSV-B isolates were detected in those genotypes. Thirteen HRSV-B TX isolates collected during RSV epidemics 1992–93 and 1993–94 were identified in the GB4 genotype cluster. The differential grouping of the BA genotype strains from GB3 genotype viruses, as described by Trento et al [17], [20], was not achieved. All HRSV-B samples collected during the 2004–05 epidemic (N = 7) clustered close to strains previously described as BA, but a bootstrap value greater than 75% was not achieved. As expected, the nucleotide analysis of these 7 strains confirmed the presence of the 60-nt duplication in the second hypervariable region of G gene. Five HRSV-B TX viruses isolated in 1993 and 1994 grouped within the GB3 viruses. Finally, 2 HRSV-B TX strains from 1993 formed an independent main lineage not related with other known genotypes (B-TX-60823-Jan93 and B-TX-64817-Dec93).

**Figure 3:**
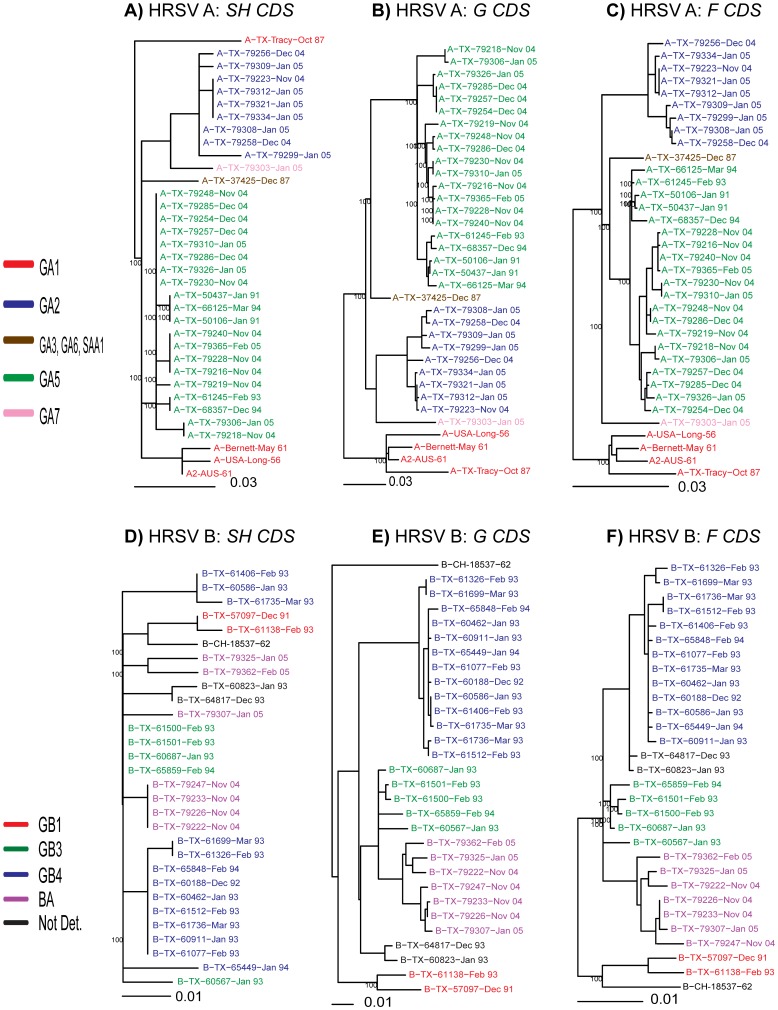
Phylogenetic trees of HRSV -A and -B, based on Coding Sequences (CDS) of SH, G and F genes. Phylogenetic tree construction was performed by maximum likelihood method and bootstrap values were calculated to support clustering. Only bootstrap values greater than 75% are shown. Colors of strains correspond to genotype classification shown in Figure 2. A), B) and C): Coding sequences of SH, G and F genes respectively, of HRSV-A strains. D), E) and F): Coding sequences of SH, G and F genes respectively, of HRSV-B strains.

Phylogenetic trees were next constructed for the coding sequences (nucleotide sequences between AUG and stop codon) of the SH, G and F genes (Figure 3) and colored according to their assigned genotype derived using the tree and group structure determined from analysis using only the distal hypervariable fragment of the G gene (Figure 2). The SH CDS sequences (Figure 3A) of TX HRSV-A produced a similar phylogenetic tree to the hypervariable fragment of the G gene CDS except for the GA1 strains which were divided into two distinct branches with RSV-A-Tracy-Oct 89 appearing as an outgroup. As shown in figure 3B, the G gene CDS of TX HRSV-A samples produced a similar tree to that obtained with the distal hypervariable fragment of G gene. Groups GA2 and GA5 were recognized and supported by 100% bootstrapping value, and the prototype strains clustered in the GA1 group. A phylogenetic tree constructed with the F gene CDS sequences of TX HRSV-A (Figure 3C) was similar to the clustering produced with the G gene CDS.

In the case of the TX HRSV-B isolates, the phylogenetic tree of SH gene CDS (Figure 3D) did not have the expected branches. The GB1 isolates clustered together while the GB4, GB3 and BA strains were grouped in non-distinct branches, although the tree was poorly resolved. The phylogenetic tree of the G gene CDS (Figure 3E) of TX HRSV-B resulted in a well define clustering of genotypes GB1, GB3, GB4 and BA. As expected, BA strains clustered close to GB3, and HRSV B-CH-18537-62 was located alone in a separate branch. The phylogenetic tree of the F gene CDS of TX HRSV-B (Figure 3F) had differences compared to the phylogenetic trees constructed with the G gene CDS (Figure 3E) and with the distal hypervariable domain (Figure 2B). The HRSV B-CH-18537-62 strain clustered with GB1 genotype isolates with a 100% bootstrapping value. The BA strains were more distant to GB3 isolates compared to the grouping observed with the G gene CDS and with the 270 nt fragment of the G gene as measured by the total number of changes.

### Nucleotide sequence analysis by genes and functional domains

Multiple alignments showed different evolutionary distances for the SH, G and F genes for the TX HRSV-A and HRSV-B isolates. It confirmed that the G gene was the most variable. The mean of distances for the SH, G and F CDS of TX HRSV-A isolates were 0.024, 0.057 and 0.026 respectively (Wilcoxon p<0.001) as measured in the mean number of differences per site. The mean of distances for the TX HRSV-B strains were 0.015, 0.041 and 0.012 for the SH, G and F gene CDS, respectively (Wilcoxon p<0.001).

#### SH gene

**Figure 4:**
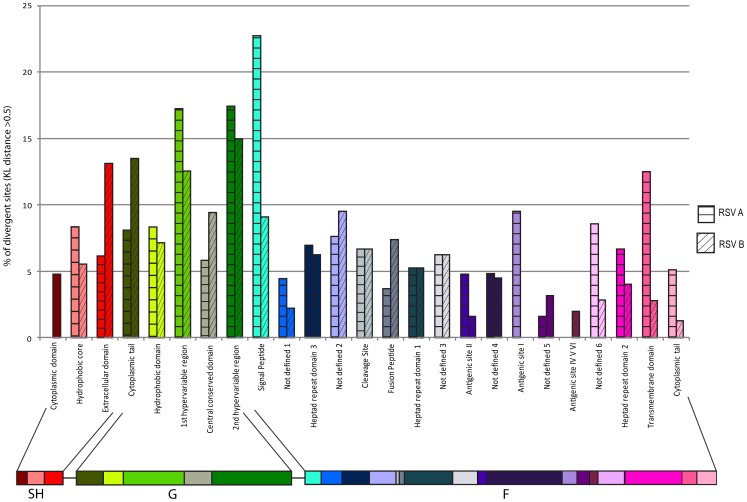
Nucleotide variability according to domains in the SH, G and F genes. KL distances were calculated for each position by the comparison between genotype GA1 to other HRSV-A strains and between reference strain B-CH-18537-62 to other HRSV-B isolates. KL values >0.5 were considered significant. Percentages of divergent sites by domains are shown for HRSV-A and HRSV-B strains.

All the TX HRSV-A samples analyzed had SH CDS of 195 nt. For TX HRSV-B isolates the SH CDS was 198 nt in length. No deletions, duplications, insertions, or alternative stop codons were detected in these sequences. Nucleotide variability was assessed by domains and pair-wise comparison was made between the prototype viruses to the TX isolates (Table 2, Figure 4). The hydrophobic core region of TX HRSV-A isolates was the most variable (6/72 sites with KL divergence >0.5), followed by the extracellular domain (5/81) and the cytoplasmic domain (0/42). For TX HRSV-B isolates the extracellular domain showed the greatest variability (11/84), followed by the hydrophobic core (4/72) and cytoplasmic domain (2/42).

**Table 2:** Nucleotide variability of the major domains of the SH, G and F genes that encode the three surface proteins of HRSV of the Texas isolates compared to the prototype viruses.

	HRSV-A			HRSV-B		
	N of Divergent sites (KL>0.5)	Nucleotides in length	% of divergent sites per functional site	N of Divergent sites (KL>0.5)	Nucleotides in length	% of divergent sites per functional site
**SH Gene**						
Cytoplasmic domain	0	42	0.00	2	42	4.76
Hydrophobic core	6	72	8.33	4	72	5.56
Extracellular domain	5	81	6.17	11	84	13.10
**G gene**						
Cytoplasmic tail	9	111	8.11	15	111	13.51
Hydrophobic domain	7	84	8.33	6	84	7.14
1^st^ hypervariable region	44	255	17.25	32	255	12.55
Central conserved domain	7	120	5.83	11	117	9.40
2^nd^ hypervariable region	57	327	17.43	49	327	14.98
**F gene**						
Signal peptide	15	66	22.73	6	66	9.09
Not defined 1	4	90	4.44	2	90	2.22
Heptad repeat domain 3	10	144	6.94	9	144	6.25
Not defined 2	8	105	7.62	10	105	9.52
Cleavage site	1	15	6.67	1	15	6.67
Fusion peptide	1	27	3.70	2	27	7.41
Heptad repeat domain 1	9	171	5.26	9	171	5.26
Not defined 3	9	144	6.25	9	144	6.25
Antigenic site II	3	63	4.76	1	63	1.59
Not defined 4	15	312	4.81	14	312	4.49
Antigenic site I	6	63	9.52	0	63	0.00
Not defined 5	1	63	1.59	2	63	3.17
Antigenic site IV	0	51	0.00	1	51	1.96
Not defined 6	9	105	8.57	3	105	2.86
Heptad repeat domain 2	10	150	6.67	6	150	4.00
Transmembrane domain	9	72	12.50	2	72	2.78
Cytoplasmic tail	4	78	5.13	1	78	1.28
*Antigenic site Øa* *	2	24	8.33	2	24	8.33
*Antigenic site Øb* *	2	45	6.66	4	45	8.88

* Not shown in Figures. Antigenic site Ø consists in two regions (called *a* and *b* in this table) structurally related in the *pre-fusion* state, and located within heptad repeat domain 3 and 1 respectively [59].

#### G gene

The length of the G gene of TX HRSV-A isolates ranged from 852 to 897 nucleotides. Two alternative stop codons caused by nucleotide substitutions in positions 850 and 892 were detected with predicted G proteins lengths of 283 (TX-A-79312-Jan05) and 297 amino acid (aa) residues (prototypes strains RSV A-Bernett-61 and A-Tracy-Oct87, plus TX-A-37425-Dec87 and A-TX-79256-Dec04). The rest of the isolates were 897 nt (298 aa) in length. Among TX HRSV-B isolates, different lengths in G CDS were detected ranging from 669 to 954 nt. As previously commented, a 6-nt in-frame-deletion was detected after position 475 in four of the 7 BA isolates collected from epidemic season 2004–2005. Also a 60 nt-duplication was detected after position 780 in all seven BA isolates collected in the 2004–05 RSV season. In addition, three alternative stop codons were identified. First, a single deletion after nucleotide 584 and a 2 nt-insertion after position 591 resulted in the presence of a premature TGA codon after the frameshift. Thus, two isolates from 1992–1993 epidemic had predicted G proteins of 222 and 223 aa, respectively (TX-B-60911-Jan93 and TX-B-61326-Feb93). Prototype RSV-B-CH-18537-62, plus 3 other isolates from 2004 had a premature stop codon due to a substitution in position 877 and 823. The predicted lengths of these proteins were 292 aa for the prototype RSV-B virus and 310 aa for the other (TX-B-79226-Nov04, TX-B-79233-Nov04 and TX-B-79307-Jan05). The last alternative stop codon and the most frequent in our isolates led to protein lengths of 295 and 315 aa. Only two of the TX HRSV-B strains used the originally described stop codon in the reference B1 strain (accession number NC_001781) resulting in 299 and 317 aa residues in length (TX-B-57097-Dec91 and TX-B-79247-Nov04).

The analysis of nucleotide variability by domains is shown in Table 2 and Figure 4. As expected, the first and second hypervariable regions of the G gene have high nt variability (>10%) in both the TX HRSV-A and HRSV-B isolates. The pattern in variation of the domains was comparable between RSV-A and RSV-B groups, although TX HRSV-B isolates also had high nt variation in the cytoplasmic tail domain.

#### F gene

All the TX HRSV-A and HRSV-B isolates had an F CDS of 1725 nt in length. No insertions, deletions, duplications, or alternative stop codons were detected in the F CDS giving a predicted length of 574 aa for all isolates. Nucleotide variability according to domains is shown in Table 2 and Figure 4. Most of the domains in the F gene of TX HRSV-A and HRSV-B isolates have nt variability <10%. The exception were the signal peptide and transmembrane domains of the TX HRSV-A isolates with nt variability as high or higher than that detected in the hypervariable domains of the G gene. Also the antigenic domains (I, II, IV and Ø) of the F gene have similar or lower nt variability compared to the central conserved domain of the G gene for both HRSV-A and HRSV-B viruses.

### Amino acid variation by genes and functional domains of HRSV strains

**Figure 5:**
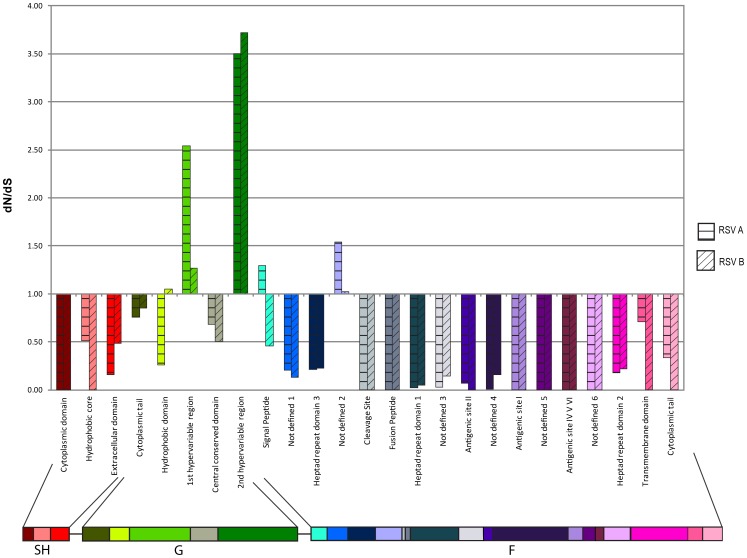
Amino acid variability according to domain in the SH, G and F genes. The non-synonymous/synonymous substitution ratio (Dn/Ds) was calculated for each domain by the comparison between GA1, GA2 and GA5 genotype strains for HRSV-A and between GB3, GB4 and BA HRSV-B isolates. Ratio values >1 are showing selection for variation between prototype (GA1) and contemporary viruses for HRSV-A and between dominant (BA) and contemporary viruses for HRSV-B.

The relative non-synonymous/synonymous substitution ratio (Dn/Ds) were calculated for each domain by the comparison between HRSV-A strains and HRSV-B strains (Table 3, Figure 5) using all pairwise comparisons of samples. For the pairwise comparison analysis, the HRSV-A prototype (GA1) viruses were included in addition to strains of contemporary genotypes. For the HRSV-B, the BA viruses (dominant genotype worldwide) and viruses from other contemporary genotypes were included. Ratio values >1 are considered to identify codons with variation. 33 HRSV-A strains were analyzed; 4 GA1, 9 GA2 and 20 GA5. In total there were 296 pair-wise comparisons per codon. 25 HRSV-B strains were analyzed; 5 GB3, 13 GB4 and 7 BA. In total there were 191 pair-wise comparisons made per codon. Most of the nt variability resulted in synonymous aa changes in the HRSV-A and HRSV-B isolates. A few domains appeared to be enriched for sites under selection for variation. These selection for variation domains in the HRSV-A isolates were the first and second hypervariable domains of G protein, and the signal peptide and a “not-defined 2” site of the F protein. The domains under selection for variation of the HRSV-B strains were the hydrophobic domain and the first and second hypervariable domains of the G protein as well as the “not-defined 2” site on the F protein. The majority of antigenic sites of the F protein for the HRSV-A and HRSV-B isolates were well conserved with Dn/Ds ratio ≤0.07. Interestingly, for the recently described antigenic site Ø [56]–[59], which is a quaternary structure consisting of two regions related in the F trimer (called Øa and Øb in this manuscript), and located within heptad repeat domains 3 and 1 respectively, higher variability was found. The antigenic region Øa of HRSV-A contemporary strains varied greatly from the HRSV-A prototype viruses with Dn/Ds ratios of infinity (all 167 aa changes identified in the clinical HRSV-A isolates were non-synonymous compared to the prototype GA1 viruses). A neutral selection in the HRSV-B isolates with a Dn/Ds ratio of 0.9.

**Table 3:** Amino acid variability by domain in the SH, G and F genes of the Texas HRSV isolates.

	HRSV-A					HRSV-B				
Genes Genetic domain (residues range)	Residues (N)	Pair-wise comparisons (N)	Syn. changes	Non-syn. changes	Non-syn/syn ratio (Dn/Ds)	Residues (N)	Pair-wise comparisons (N)	Syn. changes	Non-syn. changes	Non syn/syn ratio (Dn/Ds)
**SH Gene**										
Cytoplasmic domain (1–14)	14	4144	216	0	0.00	14	2674	150	0	0.00
Hydrophobic core (15–38)	24	7104	644	329	0.51	24	4584	86	0	0.00
Extracellular domain (39- end)	26	7696	692	108	0.16	27	5157	199	96	0.48
**G Gene**										
Cytoplasmic tail (1–37)	37	10952	716	542	0.76	37	7067	242	206	0.85
Hydrophobic domain (38–65)	28	8288	1032	267	0.26	28	5348	40	42	1.05
1^st^ hypervariable region (66–150)	85	25160	1927	4890	2.54	85	16235	653	828	1.27
Central conserved domain(151–190)	40	11840	788	537	0.68	40	7640	426	214	0.50
2^nd^ hypervariable region (191-end)	108	31968	1947	6815	3.50	108	20628	853	3174	3.72
**F Gene**										
Signal peptide (1–22)	22	6512	671	870	1.30	22	4202	174	80	0.46
Not defined 1 (23–52)	30	8880	402	81	0.20	30	5730	282	36	0.13
Heptad repeat domain 3 (53–100)	48	14208	913	196	0.21	48	9168	264	60	0.23
Not defined 2 (101–135)	35	10360	566	873	1.54	35	6685	48	49	1.02
Cleavage site (136–140)	5	1480	126	0	0.00	5	955	20	0	0.00
Fusion peptide (141–149)	9	2664	42	0	0.00	9	1719	38	0	0.00
Heptad repeat domain 1 (150–206)	57	16872	1910	42	0.02	57	10887	640	32	0.05
Not defined 3 (207–254)	48	14208	1036	29	0.03	48	9168	518	73	0.14
Antigenic site II (255–275)	21	6216	187	13	0.07	21	4011	72	0	0.00
Not defined 4 (276–379)	104	30784	3163	29	0.01	104	19864	450	72	0.16
Antigenic site I (380–400)	21	6216	630	186	0.00	21	4011	0	0	0.00
Not defined 5 (401–421)	21	6216	307	0	0.00	21	4011	0	0	0.00
Antigenic site IV (422–438)	17	5032	416	0	0.00	17	3247	174	0	0.00
Not defined 6 (439–473)	35	10360	1348	0	0.00	35	6685	354	0	0.00
Heptad repeat domain 2 (474–523)	50	14800	1581	276	0.17	50	9550	709	153	0.22
Transmembrane domain (525–548)	24	7104	699	494	0.71	24	4584	40	0	0.00
Cytoplasmic tail (550-end)	26	7696	673	224	0.33	27	5157	176	0	0.00
*Antigenic site Øa* * *(62–69)*	8	2368	0	167	∞	8	1528	20	18	0.90
*Antigenic site Øb* * *(196–210)*	15	4440	353	0	0.00	15	2865	426	32	0.08

The non-synonymous/synonymous substitution ratio (Dn/Ds) was calculated for each domain by the comparison between GA1, GA2 and GA5 genotype strains for HRSV-A and between GB3, GB4 and BA for HRSV-B isolates. Ratio values >1 are showing selection for variation between prototype (GA1) and contemporary viruses for HRSV-A and between dominant (BA) and contemporary viruses for HRSV-B.

* Not shown in Figures. Antigenic site Ø consists in two regions (called *a* and *b* in this table) structurally related in the *pre-fusion* state, and located within heptad repeat domain 3 and 1 respectively [59].

Also observed were divergent trends in the Dn/Ds ratios in five of the SH, G and F domains between HRSV-A and HRSV-B strains. The domains were the hydrophobic core of the SH protein, the hydrophobic domain of the G protein, the signal peptide of the F protein, the “not-defined 2” site of the F protein and the transmembrane domain of the F protein.

## Discussion

HRSV infections contribute to the worldwide burden of respiratory morbidity and mortality [1], [2], [4]. In recent years, viral genetic characteristics have been partially explored primarily for genotyping the strains that co-circulate during outbreaks [11]–[22]. The molecular epidemiology of HRSV is based on the phylogeny of the second hypervariable region of G gene [10]. To our knowledge, this is the first description of a genomic analysis of the three surface proteins of HRSV-A and HRSV-B isolates collected at a single site during an 18 year period. As expected from the literature, most of TX HRSV isolates clustered in well-defined genotypes; GA2 and GA5 for the HRSV-A isolates and GB4 and BA for the HRSV-B isolates, based on the second hypervariable region of the G gene. Isolates belonging to the genotype of the prototype HRSV-A and HRSV-B viruses were infrequently detected (3/60); none were identified after 1993 in our TX collection. Moreover, the prototype genotypes, clustered distant to most of the recent circulating strains. This is relevant since prototype HRSV-A viruses have traditionally been used in formulation of live and subunit candidate vaccines [60]–[62]. We consider that formulation of future candidate RSV vaccines and evaluation of new therapeutics will need to be cognizant of the genetic variation in contemporary HRSV isolates compared to the prototype laboratory strains.

The coding sequences of the SH, G and F genes were used to construct phylogenetic trees. We predicted that the phylogenetic tree constructed with the CDS of the G gene would generate clusters comparable to the genotypes that were generated with only the second hypervariable region of the G gene, while the phylogenetic trees constructed with the SH and F CDS might not cluster in patterns equivalent to their respective G-fragment based genotypes. Interestingly, the analyses revealed that the G and F CDS of HRSV-A and HRSV-B generated topologies similar to their genotypes. The phylogenetic tree constructed with the SH CDS of the HRSV-A isolates was consistent with its G-fragment based genotype topology, however, that was not true for the SH CDS of the HRSV-B isolates. This is an interesting observation that a 270 nucleotide sequence of the second hypervariable region of the G gene is predictive of the phylogenetic topologies that are formed with the full coding sequence of the G and F genes and to an extent with the SH gene.

After the analysis of a 3,500 bp fragment, we detected 100% homology in one pair of HRSV-A strains and in three pairs of HRSV-B, different from previous reports when genotyping the 270 bp fragment they found a higher frequency of identical strains [10], [16], [20], [23], [63], [64]. Although genotyping the second hypervariable region of the G gene has generated epidemiologically valuable data, we must consider that genetic variation may also be occurring in different regions, especially with variants or genotypes that consistently are associated with a greater virulence or survival advantages. Similar in concept to gene polymorphisms in the human genome, genetic variation in the genes encoding the proteins of HRSV is likely to have profound virological or biological effects on disease pathogenesis and might improve survival in the host by avoiding early recognition by the adaptive arm of the immune system.

The molecular epidemiology of HRSV was developed around the G gene based on the largest antigenic and genetic differences between HRSV -A and -B groups [8] and because it is the target of many neutralizing and protective antibodies [26]. The hypervariable domains of the G protein has a higher non-synonymous variation, indicative of a selection for variation by immune pressure, but no specific role has been attributed yet to these “mucin-like” conformations. Moreover, other functional regions of the G protein have been described as relevant in the pathogenesis of the infection. The F protein, a major protein targeted by the host's immune system [65] is antigenically conserved in the post-fusion F for both HRSV-A and HRSV-B strains. Interestingly, the recently described antigenic site Ø in the pre-fusion F appeared to be variable, especially the contemporary HRSV-A strains from the prototype viruses. Monoclonal antibodies generated to antigenic site Ø appear to have greater neutralizing capacity compared to palivizumab, a monoclonal antibody that targets the highly conserved antigenic site II [58]. The high variability of antigenic site Ø detected in the clinical HRSV-A isolates raises caution to its potential as a vaccine target.

Insufficient information is available on the SH protein. To further address the potential variability of the surface proteins, the nucleotide and amino acid CDS for the SH, G, and F genes were evaluated. According to our genetic variability analysis, it was confirmed that the G gene was the most variable, with the SH and F genes having lower and comparable means of distances for the HRSV-A strains and HRSV-B strains.

Genetic variability of the Texas isolates was further explored by evaluating nucleotide divergence from the prototype viruses based on known functional sites or domains on the SH, G and F genes. As expected, the first and second hypervariable domains on the G gene of the TX strains in both HRSV groups had greater than 10% of divergent sites compared to the prototype strains. Remarkably, the HRSV-B TX strains, the central conserved region demonstrated nucleotide divergence approaching 10% and the cytoplasmatic tail domain showed nucleotide variability higher than the first hypervariable domain. The central conserved region of the G protein is being evaluated for its potential as a vaccine candidate [66] and the cytoplasmatic tail has been studied for its possible role in eliciting the innate immune response [29]. On the F gene, the signal peptide and transmembrane domains of the TX isolates of the HRSV-A group demonstrated significant divergence compared to the prototype strains (more than 10% and 15% respectively). Antigenic site II of the F protein is the antigenic target of palivizumab, a highly effective humanized monoclonal antibody used in the immunoprophylaxis of high risk infants [5]. Nucleotide sequences of the antigenic site II of the TX strains appeared well conserved and comparable to the respective prototype HRSV-A and HRSV-B viruses. The nucleotide sequences of antigenic site I of the HRSV-A strains appeared more divergent compared to the other antigenic domains of the F gene. To our knowledge, there were 6 domains (“Not-defined” 1 to 6) on the F gene for which no function or antigenic activity have been described. . Interestingly, Not-defined 2 domain on the HRSV-A and HRSV-B isolates and Not-define 6 domain in the HRSV-A strains had nucleotide divergence between 5 to 10%. Their significance is unknown at this time. Heptad repeat domain 1 and heptad repeat domain 2 are target sites for fusion inhibitor compounds [67]. Both of these heptad repeat domains had nucleotide divergence of approximately 5% for the HRSV-A and HRSV-B TX isolates compared to the prototype strains. Finally, in the case of SH gene, the extracellular domain and hydrophobic core demonstrated the higher frequencies of divergent sites. This study confirms that nucleotide variability is not uniform across the different functional domains of the three surface proteins of HRSV. Moreover, some domains with unknown function are more conserved than others. Our method of performing the selection for variation analysis was based on a direct assessment of non-synonymous and synonymous events per codon within each protein, and the same computational method was applied equally to each codon across each protein sequence. The cross tabulation analysis within each protein was then determined by overlay of functional annotation of the regions. Our approach differs from other approaches such as the SLAC method which attempts to determine p-values for positive selection for each codon. Analysis using SLAC method did not detect significant evidence for positively selected sites in our TX HRSV isolates. Still, we believe the direct comparative approach we have taken is useful as a relative assessment of the character of variation across each protein compared to prototype viruses.

Most of these nucleotide changes did not result in amino acid changes. As expected, first and second hypervariable regions of the G gene showed high selection for variation from the prototype viruses, and as mentioned, post-fusion antigenic sites on the F gene demonstrated high conservation between genotypes. However, the newly defined antigenic site Ø in the pre-fusion F appeared variable especially among contemporary HRSV-A strains compared to prototype viruses. Interestingly, five domains on the SH, G and F genes demonstrated site divergence between HRSV -A and -B strains: hydrophobic core of SH, hydrophobic domain in G gene, signal peptide of F, transmembrane domain of F, and “Not defined 2” site in the F gene. To our knowledge, none of these regions have been previously studied regarding their possible functional implications in disease or eventual treatments. The non-synonymous to synonymous ratios for the heptad repeat domains were consistent with domain conservation (Dn/Ds<1). Fusion inhibitor molecules directed to these domains are entering clinical trials. It will be important to determine if breakthrough infections are the result of selective pressure in the heptad repeat domain regions and whether viral fitness is altered.

Novel information was gained by analyzing the genes of the three surface proteins of HRSV strains that circulated in Houston, TX over a 18 year period. It will be relevant to determine their variation in different areas worldwide. We hypothesize that an analysis beyond genotype classification is needed because some of these genomic differences might impact the biology of infection and disease by providing the virus with possible survival advantage in a partially immune-protected community. This novel approach, describing genome characteristics according to functional domains, can give us some clues in the understanding of the genetic contribution to human disease from the virus perspective.

## Supporting Information

Table S1 HRSV gene sequences retrieved from Genbank: 62 HRSV-A and 53 HRSV-B unique isolates.
(DOC)
