# Supplementary material for: Gene Sequence Variability of the Three Surface Proteins of Human Respiratory Syncytial Virus (HRSV) in Texas
Source: PLoS One. 2014 Mar 13;9(3):e90786. doi: 10.1371/journal.pone.0090786 (PMC3953119; doi:10.1371/journal.pone.0090786)
Supplement: Table S1 — HRSV gene sequences retrieved from Genbank: 62 HRSV-A and 53 HRSV-B unique isolates. (DOC) [file pone.0090786.s001.doc]

| **Virus Strain** | **Genbank ID N#** | **Genotype** | **Reference** |
| --- | --- | --- | --- |
|  |  |  |  |
| A-Ab4026B01-G-FRAG | AY146435 | GA2 | Madhi S. J Clin Virol 2003; 27:180-189 |
| A-Ab5076Pt01-G-FRAG | AY146437 | GA5 | Madhi S. J Clin Virol 2003; 27:180-189 |
| A-AL19376-1-G-FRAG | AF233900 | GA2 | Peret T. JID 2000; 181:1891-1896 |
| A-AL19452-2-G-FRAG | AF233901 | GA6 | Peret T. JID 2000; 181:1891-1896 |
| A-AL19471-5-G-FRAG | AF233902 | GA1 | Peret T. JID 2000; 181:1891-1896 |
| A-AL19556-3-G-FRAG | AF233903 | GA5 | Peret T. JID 2000; 181:1891-1896 |
| A-CH09-G-FRAG | AF065254 | GA4 | Peret T. J Gen Virol 1998; 79:2221-2229 |
| A-CH17-G-FRAG | AF065255 | GA5 | Peret T. J Gen Virol 1998; 79:2221-2229 |
| A-CH28-G-FRAG | AF065256 | GA2 | Peret T. J Gen Virol 1998; 79:2221-2229 |
| A-CH34-G-FRAG | AF065257 | GA1 | Peret T. J Gen Virol 1998; 79:2221-2229 |
| A-CN1973-G-FRAG | AF233904 | GA7 | Peret T. JID 2000; 181:1891-1896 |
| A-CN2395-G-FRAG | AF233905 | GA3 | Peret T. JID 2000; 181:1891-1896 |
| A-CN2708-G-FRAG | AF233906 | GA5 | Peret T. JID 2000; 181:1891-1896 |
| A-CN2851-G-FRAG | AF233907 | GA7 | Peret T. JID 2000; 181:1891-1896 |
| A-MAD-3-92-G-FRAG | Z33455 | GA7 | Garcia O. J Virol 1994; 68:5448-5459 |
| A-MAD-5-92-G-FRAG | Z33417 | GA7 | Garcia O. J Virol 1994; 68:5448-5459 |
| A-MO01-G-FRAG | AF233909 | GA5 | Peret T. JID 2000; 181:1891-1896 |
| A-MO02-G-FRAG | AF233910 | GA7 | Peret T. JID 2000; 181:1891-1896 |
| A-MO16-G-FRAG | AF233913 | GA3 | Peret T. JID 2000; 181:1891-1896 |
| A-MO48-G-FRAG | AF233914 | GA1 | Peret T. JID 2000; 181:1891-1896 |
| A-MO55-G-FRAG | AF233915 | GA2 | Peret T. JID 2000; 181:1891-1896 |
| A-MON-1-90-G-FRAG | Z33494 | GA5 | Garcia O. J Virol 1994; 68:5448-5459 |
| A-MON-3-88-G-FRAG | Z33425 | GA2 | Garcia O. J Virol 1994; 68:5448-5459 |
| A-MON-4-90-G-FRAG | Z33426 | GA3 | Garcia O. J Virol 1994; 68:5448-5459 |
| A-MON-5-90-G-FRAG | Z33427 | GA1 | Garcia O. J Virol 1994; 68:5448-5459 |
| A-MON-8-92-G-FRAG | Z33430 | GA5 | Garcia O. J Virol 1994; 68:5448-5459 |
| A-MON-9-91-G-FRAG | Z33431 | GA1 | Garcia O. J Virol 1994; 68:5448-5459 |
| A-MON-9-92-G-FRAG | Z33432 | GA1 | Garcia O. J Virol 1994; 68:5448-5459 |
| A-NY20-G-FRAG | AF233918 | GA6 | Peret T. JID 2000; 181:1891-1896 |
| A-NY103-G-FRAG | AF233916 | GA5 | Peret T. JID 2000; 181:1891-1896 |
| A-NY108-G-FRAG | AF233917 | GA1 | Peret T. JID 2000; 181:1891-1896 |
| A-SA97D1289-97-G-FRAG | AF348803 | GA5 | Venter M. J General Virol 2001; 82: 2117-2124 |
| A-SA98V603-98-G-FRAG | AF348807 | SAA1 | Venter M. J General Virol 2001; 82: 2117-2124 |
| A-SA99V360-99-G-FRAG | AF348804 | GA7 | Venter M. J General Virol 2001; 82: 2117-2124 |
| A-SA99V1239-99-G-FRAG | AF348808 | SAA1 | Venter M. J General Virol 2001; 82: 2117-2124 |
| A-Sal-87-99-G-FRAG | AY472086 | GA2 | Moura F. J Med Virol 2004; 74:156-160 |
| A-Sal-173-99-G-FRAG | AY472094 | GA5 | Moura F. J Med Virol 2004; 74:156-160 |
| A-BA-2155-96-G-FRAG | AY667071 | GA3 | Galiano M. J Clin Microbiol 2005; 43: 2266-2273 |
| A-BA-5948-01-G-FRAG | AY667093 | GA2 | Galiano M. J Clin Microbiol 2005; 43: 2266-2273 |
| A-BA-5046-00-G-FRAG | AY667089 | GA2 | Galiano M. J Clin Microbiol 2005; 43: 2266-2273 |
| A-BA-3144-98-G-FRAG | AY667079 | GA2 | Galiano M. J Clin Microbiol 2005; 43: 2266-2273 |
| A-BA-3793-99-G-FRAG | AY667081 | GA2 | Galiano M. J Clin Microbiol 2005; 43: 2266-2273 |
| A-BA-1-96-G-FRAG | AF516115 | GA1 | Frabasile S. J Med Virol 2003; 71:305-312 |
| A-BA-2-96-G-FRAG | AF516116 | GA1 | Frabasile S. J Med Virol 2003; 71:305-312 |
| A-LLC235-267-G-FRAG | AY114149 | GA2 | Lim C. Acta Virol 2003;47(2):97-104 |
| A-BE-11030-00-G-FRAG | AY343599 | GA2 | Zlateva K. J Clin Microbiol 2007; 45: 3022-3030 |
| A-BE-12511-96-G-FRAG | DQ985118 | GA2 | Zlateva K. J Clin Microbiol 2007; 45: 3022-3030 |
| A-BE-8078-92-93-G-FRAG | AY343657 | GA4 | Zlateva K. J Clin Microbiol 2007; 45: 3022-3030 |
| A-BE-12243-96-G-FRAG | AY343647 | GA2 | Zlateva K. J Clin Microbiol 2007; 45: 3022-3030 |
| A-WI-629-2-07-G-FRAG | JF920046 | GA2 | Rebuffo-Scheer C. PLoS ONE 2011; 6(10): e25468 |
| A-WI-629-23-08-G-FRAG | JF920047 | GA2 | Rebuffo-Scheer C. PLoS ONE 2011; 6(10): e25468 |
| A-WI-629-9-2-07-G-FRAG | JF920048 | GA2 | Rebuffo-Scheer C. PLoS ONE 2011; 6(10): e25468 |
| A-WI-629-DC9-08-09-G-FRAG | JF920050 | GA2 | Rebuffo-Scheer C. PLoS ONE 2011; 6(10): e25468 |
| A-WI-629-22-07-G-FRAG | JF920049 | GA2 | Rebuffo-Scheer C. PLoS ONE 2011; 6(10): e25468 |
| A-WI-629-21-07-G-FRAG | JF920051 | GA2 | Rebuffo-Scheer C. PLoS ONE 2011; 6(10): e25468 |
| A-WI-629-Q0284-10-G-FRAG | JF920053 | GA2 | Rebuffo-Scheer C. PLoS ONE 2011; 6(10): e25468 |
| A-WI-629-Q0154-10-G-FRAG | JF920052 | GA2 | Rebuffo-Scheer C. PLoS ONE 2011; 6(10): e25468 |
| A-WI-629-Q0282-10-G-FRAG | JF920054 | GA2 | Rebuffo-Scheer C. PLoS ONE 2011; 6(10): e25468 |
| A-WI-629-17-06-07-G-FRAG | JF920058 | GA5 | Rebuffo-Scheer C. PLoS ONE 2011; 6(10): e25468 |
| A-WI-629-3248-98-G-FRAG | JF920062 | GA7 | Rebuffo-Scheer C. PLoS ONE 2011; 6(10): e25468 |
| A-WI-629-4071-98-G-FRAG | JF920065 | GA7 | Rebuffo-Scheer C. PLoS ONE 2011; 6(10): e25468 |
| A-WI-629-3-06-07-G-FRAG | JF920069 | GA1 | Rebuffo-Scheer C. PLoS ONE 2011; 6(10): e25468 |
| A-WI-629-4239-98-G-FRAG | JF920057 | GA2 | Rebuffo-Scheer C. PLoS ONE 2011; 6(10): e25468 |
|  |  |  |  |
| B-AL19734-4-G-FRAG | AAF75974 | GB 4 | Peret T. JID 2000; 181:1891-1896 |
| B-AL19794-1-G-FRAG | AAF75975 | GB 3 | Peret T. JID 2000; 181:1891-1896 |
| B-BA-1004-02-G-FRAG | DQ227376 | BA IV | Trento A. J Virol 2006; 80: 975-984. |
| B-BA-164-02-G-FRAG | DQ227367 | BA I | Trento A. J Virol 2006; 80: 975-984. |
| B-BA-354-04-G-FRAG | DQ227406 | BA IV | Trento A. J Virol 2006; 80: 975-984. |
| B-BA-493-04-G-FRAG | DQ227407 | BA IV | Trento A. J Virol 2006; 80: 975-984. |
| B-BA-770-02-G-FRAG | DQ227373 | BA I | Trento A. J Virol 2006; 80: 975-984. |
| B-BA-1441-02-G-FRAG | DQ227381 | BA III | Trento A. J Virol 2006; 80: 975-984. |
| B-BA-2574-97-G-FRAG | AY672688 | GB 3 | Galiano M. J Clin Microbiol 2005; 43:2266-2273 |
| B-BA-2960-98-G-FRAG | AY672690 | GB 3 | Galiano M. J Clin Microbiol 2005; 43:2266-2273 |
| B-BA-3018-98-G-FRAG | AY672691 | GB 4 | Galiano M. J Clin Microbiol 2005; 43:2266-2273 |
| B-BA-3768-99-G-FRAG | AY672693 | SAB-3 | Galiano M. J Clin Microbiol 2005; 43:2266-2273 |
| B-BA-3833-99-G-FRAG | AY333362 | BA I | Trento A. J General Virol 2003; 84:3115-3120 |
| B-BA-3910-99-G-FRAG | AY672696 | SAB-3 | Galiano M. J Clin Microbiol 2005; 43:2266-2273 |
| B-BA-3923-99-G-FRAG | AY672697 | GB 3 | Galiano M. J Clin Microbiol 2005; 43:2266-2273 |
| B-BA-3976-99-G-FRAG | AY672698 | GB 4 | Galiano M. J Clin Microbiol 2005; 43:2266-2273 |
| B-BA-4062-99-G-FRAG | AY672699 | GB 3 | Galiano M. J Clin Microbiol 2005; 43:2266-2273 |
| B-BA-4852-03-G-FRAG | DQ227403 | BA III | Trento A. J Virol 2006; 80: 975-984. |
| B-BA-5021-03-G-FRAG | DQ227405 | BA III | Trento A. J Virol 2006; 80: 975-984. |
| B-BA-5954-01-G-FRAG | AY672700 | GB3 | Galiano M. J Clin Microbiol 2005; 43:2266-2273 |
| B-BA-5997-01-G-FRAG | AY672701 | SAB-3 | Galiano M. J Clin Microbiol 2005; 43:2266-2273 |
| B-BE-1066-03-G-FRAG | AY751123 | BA II | Zlaveta K. J Virol 2005; 79:9157-9167 |
| B-BE-11508-02-G-FRAG | AY751116 | BA VI | Zlaveta K. J Virol 2005; 79:9157-9167 |
| B-BE-11535-01-G-FRAG | AY751126 | BA I | Zlaveta K. J Virol 2005; 79:9157-9167 |
| B-BE-12445-99-G-FRAG | AY751094 | BA IV | Zlaveta K. J Virol 2005; 79:9157-9167 |
| B-BE-12522-01-G-FRAG | AY751119 | BA II | Zlaveta K. J Virol 2005; 79:9157-9167 |
| B-BE-14610-03-G-FRAG | AY751093 | BA IV | Zlaveta K. J Virol 2005; 79:9157-9167 |
| B-CH10b-90-G-FRAG | AF065250 | GB 1 | Peret T. J Gen Virol 1998; 79:2221-2229 |
| B-CH93-9b-92-G-FRAG | AF065251 | GB 2 | Peret T. J Gen Virol 1998; 79:2221-2229 |
| B-CH93-18b-92-G-FRAG | AF065252 | GB 3 | Peret T. J Gen Virol 1998; 79:2221-2229 |
| B-CH93-53b-93-G-FRAG | AF065253 | GB 3 | Peret T. J Gen Virol 1998; 79:2221-2229 |
| B-CN1839-G-FRAG | AAF75976 | GB 4 | Peret T. JID 2000; 181:1891-1896 |
| B-MAD-1-91-G-FRAG | CQ144963 | GB 2 | Martinez I. J Gen Virol 1999; 80: 125-130 |
| B-MO30-G-FRAG | AAF75978 | GB 4 | Peret T. JID 2000; 181:1891-1896 |
| B-MO35-G-FRAG | AAF75979 | GB 3 | Peret T. JID 2000; 181:1891-1896 |
| B-MO53-G-FRAG | AAF75980 | GB 3 | Peret T. JID 2000; 181:1891-1896 |
| B-MON-8-01-G-FRAG | AY488805 | URU-1 | Blanc A. Arch Virol 2005; 150:603-609 |
| B-MON-9-01-G-FRAG | AY488806 | URU-2 | Blanc A. Arch Virol 2005; 150:603-609 |
| B-Moz-198-99-G-FRAG | AF309676 | SAB 2 | Roca A. J Gen Virol 2001; 82: 103-111 |
| B-NG-004-03-G-FRAG | AB175819 | BA V | Sato M. J Clin Microbiol 2005; 43:36-40 |
| B-NG-006-03-G-FRAG | AB175820 | BA V | Sato M. J Clin Microbiol 2005; 43:36-40 |
| B-NG-153-03-G-FRAG | AB175821 | BA II | Sato M. J Clin Microbiol 2005; 43:36-40 |
| B-NY01-G-FRAG | AAF75981 | GB 4 | Peret T. JID 2000; 181:1891-1896 |
| B-NY97-G-FRAG | AAF75982 | GB 3 | Peret T. JID 2000; 181:1891-1896 |
| B-QUE-18-02-G-FRAG | AY927401 | BA VI | Gilca R. JID 2006; 193:54-58 |
| B-QUE-29-01-G-FRAG | AY927402 | BA IV | Gilca R. JID 2006; 193:54-58 |
| B-QUE-70-01-G-FRAG | AY927406 | BA IV | Gilca R. JID 2006; 193:54-58 |
| B-SA99V429-99-G-FRAG | AF348813 | SAB 3 | Venter M. J General Virol 2001; 82: 2117-2124 |
| B-SA99V800-99-G-FRAG | AF348821 | SAB 2 | Venter M. J General Virol 2001; 82: 2117-2124 |
| B-SAP-71-02-G-FRAG | AB161414 | BA VI | Nagai K. J Med Virol 2004; 74:161-165 |
| B-WI-629-5B-06-07-G-FRAG | JN032115 | BA | Rebuffo-Scheer C. PLoS ONE 2011; 6(10): e25468 |
| B-WI-629-12-06-07-G-FRAG | JN032116 | BA | Rebuffo-Scheer C. PLoS ONE 2011; 6(10): e25468 |
| B-WI-629-15-06-07-G-FRAG | JN032117 | BA | Rebuffo-Scheer C. PLoS ONE 2011; 6(10): e25468 |
| B-WI-629-DC1-08-09-G-FRAG | JN032119 | BA | Rebuffo-Scheer C. PLoS ONE 2011; 6(10): e25468 |
| B-WI-629-Q0190-10-G-FRAG | JN032120 | BA | Rebuffo-Scheer C. PLoS ONE 2011; 6(10): e25468 |
| B-BE-4618-88-G-FRAG | AY751255 | GB1 | Zlaveta K. J Virol 2005; 79:9157-9167 |
| B-BE-2968-85-G-FRAG | AY751257 | GB1 | Zlaveta K. J Virol 2005; 79:9157-9167 |
| B-WN-15291-85-G-FRAG | M73542 | GB1 | Sullender WM. J Virol 1991; 65(10):5425-34 |
